# Super enhancer lncRNAs: a novel hallmark in cancer

**DOI:** 10.1186/s12964-024-01599-6

**Published:** 2024-04-02

**Authors:** Ping Song, Rongyan Han, Fan Yang

**Affiliations:** 1 Department of Gastroenterology, Affiliated Hangzhou First People’s Hospital, Westlake University, Hangzhou, 310006 Zhejiang Province China; 2 Key Laboratory of Integrated Traditional Chinese and Western Medicine for Biliary and Pancreatic Diseases of Zhejiang Province, Hangzhou, 310006 China; 3 Hangzhou Institute of Digestive Diseases, Hangzhou, 310006 China; 4 Key Laboratory of Integrated Oncology and Intelligent Medicine of Zhejiang Province, Department of Hepatobiliary and Pancreatic Surgery, Affiliated Hangzhou First People’s Hospital, School of Medicine, Westlake University, Hangzhou, 310006 Zhejiang Province China; 5 Department of emergency, Affiliated Hangzhou First People’s Hospital, Westlake University, Hangzhou, 310006 Zhejiang Province China

**Keywords:** Super enhancer, SE-lncRNA, Cancer, BRD4, CDK

## Abstract

Super enhancers (SEs) consist of clusters of enhancers, harboring an unusually high density of transcription factors, mediator coactivators and epigenetic modifications. SEs play a crucial role in the maintenance of cancer cell identity and promoting oncogenic transcription. Super enhancer lncRNAs (SE-lncRNAs) refer to either transcript from SEs locus or interact with SEs, whose transcriptional activity is highly dependent on SEs. Moreover, these SE-lncRNAs can interact with their associated enhancer regions in cis and modulate the expression of oncogenes or key signal pathways in cancers. Inhibition of SEs would be a promising therapy for cancer. In this review, we summarize the research of SE-lncRNAs in different kinds of cancers so far and decode the mechanism of SE-lncRNAs in carcinogenesis to provide novel ideas for the cancer therapy.

## Introduction

Cancer has been recognized as a disease resulting from the accumulation of multiple genetic and epigenetic dysregulation. The high expression of oncogenes plays a crucial role in the process of carcinogenesis. High expression of oncogenes mechanistically influence cancer cell in following ways: (1) Stimulate the mRNA transcription; (2) Slow down the mRNA decay; (3) Improve protein translation efficiency; (4) Deregulate protein modification to prevent its degradation [1–4]. However, only 2% of mammalian genomes are protein-coding genes, even though > 80% of the DNA is transcribed [5]. Increasing studies have shown that noncoding RNAs play an important role in tumor progression by regulating the chromatin organization, transcription, mRNA stability, protein translation, and post-translational modification [6, 7].

Enhancers are cis-regulatory DNA regions that interact with proteins to strengthen the gene transcription [8]. Super enhancers (SEs) consist of clusters of enhancers, harboring an unusually high density of transcription factors, mediator coactivators and epigenetic modifications [9]. Furthermore, SEs span a larger genomic region with the median size ranging from 10 kb to over 60 kb and enrich histone markers (H3K27ac and H3K4me1) associated with transcription activity [10]. SEs can define a cell identity through driving the expression of oncogenes and signaling pathways [11], such as the Wnt signaling and MEK1/ERK1/2 pathway, etc [12]. These unique characteristics indicate that SEs play an important role in cancer.

**Fig. 1:**
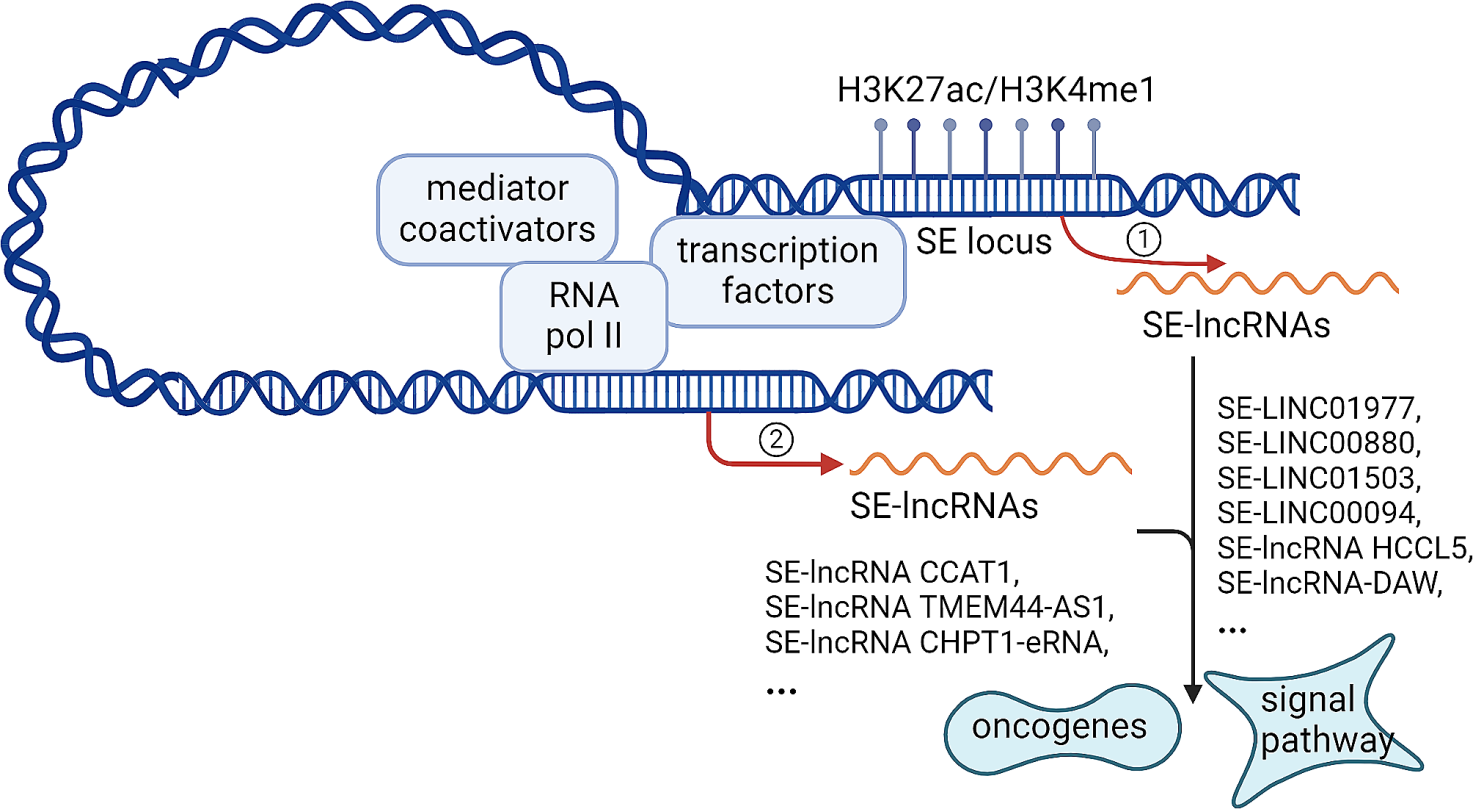
The model of SE-lncRNAs. The super enhancer lncRNAs (SE-lncRNAs) refer in particular to transcript from (1) or to interact with SEs (2), whose transcriptional activity is highly dependent on SEs. These SE-lncRNAs can interact with their associated enhancer regions in cis and modulate the expression of oncogenes or key signal pathways

Most SEs transcript RNAs (seRNAs), including microRNAs, lncRNAs, etc [13]. The super enhancer lncRNAs (SE-lncRNAs) refer in particular to transcript from or to interact with SEs, whose transcriptional activity is highly dependent on SEs (Fig. 1) [14, 15]. These SE-lncRNAs can interact with their associated enhancer regions in cis and modulate the expression of neighboring genes [16] .

Although the function of SEs and lncRNAs are well characterized, the regulation of SE-lncRNAs, especially the mechanism and function in cancer remain largely unknown. In this review, we aimed to summarize the recent advances on SE-lncRNAs in different cancers (Table 1) to facilitate a better understanding of the mechanism of carcinogenesis and offer some hints for novel approaches in precision therapy of human cancers.

**Table 1:** SE-lncRNAs in cance

SE-lncRNAs	Cancer	interact with oncogenes or pathway	Ref
LINC01977 LINC00880 LINC01503	LUAD LUAD NSCLC	TGF-β/SMAD3 pathway PI3K/AKT pathway LASP1	17 18 19
LINC01503 CCAT1 LINC00094 LINC00094	HNSCC ESCC ESCC CSCC	ERK signaling and AKT signaling TP63/SOX2-CCAT1-EGFR cascade TCF3, KLF5 MMP-1, MMP-13, collagen I	20 21 22 23
lncRNA HCCL5 LncRNA-DAW LINC01004 LINC01089 HSAL3	HCC HCC HCC HCC HCC	ZEB1 Wnt/b-catenin pathway E2F1 ERK/Elk1/Snail axis NOTCH signaling	24 12 25 26 27
CCAT1-L AC005592.2 LINC00857	CRC CRC CRC	MYC OLFM4 SLC1A5/ASCT2	28 29 30
TM4SF1-AS1	STAD	T cell-mediated immune killing function	34
RP11-379F4.4 RP11-465B22.8 DSCAM-AS1	DCIS DCIS BRCA	RARRES1 miR-200b FOXA1 and ERα apoERα, p300, GATA3, FoxM1 and CTCF	35 35 36 37
ATP1A1 − AS1	BRCA	ATP1A1	38
MANCR CHPT1-eRNA	CRPC CRPC	migration and invasion CHPT1	39 40
LINC00162	BLCA	PTTG1IP	41
LIMD1-AS1 TMEM44-AS1 LINC00945	Glioma Glioma Glioma	interferon signaling Myc and EGR1/IL-6 signaling proliferation, EMT, migration, and invasion	42 43 44
TP53TG1 LOC100506178 SUCLG2-AS1	NPC NPC NPC	malignant phenotypes MICAL2 SOX2	45 46 47

### Lung cancer

By overlapping the dysregulated lncRNA from TCGA and SE-lncRNA microarrays, which contented five paired tumor and non-tumor tissues from Lung adenocarcinoma (LUAD) patients, Zhang et al. identified and focused on the most significantly differentially expressed lncRNA, SE-LINC01977. Furthermore, ChIP-seq, Hi-C data analysis, and luciferase reporter assays were utilized to confirm the hijacking of LINC01977 by SE. SMAD3 up-regulated LINC09177 transcription by simultaneously binding the promoter and SE, which was induced by the infiltration of M2-like tumor-associated macrophages (TAM2), subsequently activating the TGF-β/SMAD3 pathway [17].

This group also found LINC00880, a SE-driven lncRNA, was higher expression in LUAD. Mechanically, the transcription factor FOXP3 could simultaneously occupy the promoter and SE regions of LINC00880 to promote its transcription. By forming a ternary complex with CDK1 and PRDX as a protein scaffold, LINC00880 increased the kinase activity of CDK1 and activated PTEN-AKT pathway [18].

LINC01503, a SE-lncRNA upregulated in non-small-cell lung cancer (NSCLC), was found influencing NSCLC cancer cell biology. LINC01503 knock-down suppressed proliferation, migration, and invasion of NSCLC cells in vitro. Mechanistically, SE-LINC01503 downregulated MiR-342-3p to facilitate LASP1 expression [19].

Lung cancer was the leading cause of cancer death globally. Studies on the epigenetic characteristics of lung cancer played an important role in diagnosis and treatment. SE-lncRNAs were novel epigenetic regulation whose function in promoting tumorigenesis and development gradually emerged. More cancer-promoting SE-lncRNAs would be discovered in the future.

### Squamous cell carcinoma (SCC)

Interestingly, the expression of SE-LINC01503 was significantly higher in head and neck squamous cell carcinoma (HNSCC) comparing to matched neighboring normal tissue, indicating poor prognosis. SE-LINC01503 enhanced the proliferation, migration and invasion of SCC cells. Mechanistically, TP63 activated the transcription of SE-LINC01503 by binding its SEs locus. As a result, SE-LINC01503 increased ERK2 phosphorylation by DUSP6 and activated ERK signaling via MAPK. On the other hand, SE-LINC01503 disrupted the interaction between EBP1 and the p85 subunit of PI3K, activating AKT signaling [20].

Based on analysis of TCGA, ChIP-seq and RNA-seq data, lncRNA CCAT1, a super-enhancer-associated gene and located at chr8q24.2 was found highly expressed in esophageal SCC (ESCC). SE-lncRNA CCAT1 was a common target of transcript factors TP63 and SOX2, which co-occupied at both the promoter and SEs of CCAT1. ChIRP analysis showed CCAT1-formed specific DNA/RNA/protein complex bound to the SEs of EGFR and increased the expression of EGFR, leading to activation of both MEK/ERK1/2 and PI3K/AKT signaling pathways [21].

By analyzing for SEs associated competing endogenous lncRNA (ce-lncRNA) in ESCC, Wang et al. demonstrated HOTAIR, XIST, SNHG5, and LINC00094. LINC00094 could be activated by transcription factors TCF3 and KLF5 through binding to SE regions and promoted the proliferation of ESCC cells [22]. Meanwhile, SE-LINC00094 was also highly expressed in cutaneous squamous cell carcinoma (CSCC). Knockdown of LINC00094 resulted in decreased expression of MMP-1 and MMP-13 and suppressed invasion of CSCC cells through collagen I [23].

With the development of advanced research techniques, the potential relationship of SE-lncRNAs and signaling pathways in the SCC would be explored and the mechanism of SE-lncRNAs would be more detailed. Inhibition of SE-lncRNAs can become one of key methods for precise therapy of SCC.

### Hepatocellular carcinoma (HCC)

SE-lncRNA HCCL5 promoted the viability, migration, and epithelial-mesenchymal transition (EMT) of HCC cells by increasing the expression of EMT-related transcription factors. By analysis of H3K27ac ChIP-seq data and ChIA-PET (Chromatin Interaction Analysis with Paired-End-Tag sequencing) data, Peng et al. identified a SE 18 kb downstream of HCCL5 which was corroborated by the co-occupancy of both pol2 and H3K4me. What’s more, ZEB1 bound to the identified SE and increased the expression of HCCL5 [24].

Based on public databases dbSUPER (https://asntech.org/dbsuper/index.php) and Cistrome (http://cistrome.org/db/#/), Liang et al. found that chromosome 20q13.12 harbored strong H3K27ac, H3K4me1 and weak H3K27me3 signal in normal hepatocytes, which indicated this locus might harbor a liver-specific SE. LncRNA-DAW was driven by this location and transcriptionally activated by transcription factor HNF4G in HCC. In addition, SE-lncRNA-DAW mediated EZH2 degradation to facilitate the transcription of Wnt2, which consequently activated the Wnt/b-catenin signaling [12].

Based on analysis of the H3K27ac ChIP-seq data from HepG2 cell line (ENCODE, ENCSR000AMO) and two HCC tissues (GEO, GSE112221), LINC01004 was identified as a novel SE-driven lncRNA, which was regulated by the transcription factor E2F1 to promote the proliferation and metastasis of HCC cell [25].

SE-lncRNA LINC01089 promoted the EMT and invasion of HCC cell by promoting the ERK/Elk1/Snail axis. In mechanism, E2F1 bound to a LINC01089 SE and promoted the transcription of LINC01089. LINC01089 interacted with hnRNPM to decrease DIAPH3 mRNA stability, whose exon 3 contained an important m6A-modification site that was recognized by IGF2BP3, thus activating the ERK/Elk1/Snail axis [26].

HSAL3 was highly expressed in HCC and correlated with poor prognosis, it was identified as an uncharacterized SE-driven oncogenic lncRNA which was activated by transcription factors HCFC1 and HSF1 via its SE. SE-lncRNA HSAL3 positively regulated NOTCH signaling in HCC [27].

HCC was the most common primary malignant liver tumor with high mortality. Epigenetic aberrations were found closely related to the proliferation and metastasis of HCC, and of the potential role of SE-lncRNAs in malignant phenotype of HCC cells has attracted many researchers.

### Colorectal cancer (CRC)

SE-lncRNA CCAT1-L was abundantly transcribed specifically in CRC from a locus 515 kb upstream of MYC on 8q24. Genome-wide mapping of enhancers, which was based on search for locations with high H3K27ac and high H3K4me1 but low H3K4me3 modification, revealed that the enomic locus encoding CCAT1-L spans up to 150 kb in length. This region was also enriched for CBP/P300 binding site. In addition, the expression of MYC reduced after CCAT1-L knockdown. With Chromosome Conformation Capture (3 C) and double DNA FISH, a well-characterized chromatin loop between a locus 335 kb upstream (MYC-335) and the MYC promoter was found with the transcription factors TCF4 and CTCF bound. Based on the loop, CCAT1-L activated MYC expression across large distances [28].

Analysis of the SE-lncRNA microarray was carried out to profile the differentially expressed SE-lncRNAs in four CRC tissues and peritumoral tissues. SE-lncRNA AC005592.2 was filtrated with highly expressed in CRC and significantly associated with poor prognosis. AC005592.2 enhanced proliferation, invasion and migration of CRC cells by promoting the expression of olfactomedin 4 (OLFM4) [29].

Analysis of H3K27ac ChIP-seq and lncRNA microarray screened out that HSF1 mediated SE-lncRNA LINC00857. HSF1 collaborated with P300 to increase the transcription of LINC00857 by its SE locus. LINC00857 contributed to SLC1A5/ASCT2-mediated glutamine transport in CRC [30].

MYC was aberrantly expressed in both tumor initiation and maintenance. The global genes involved in cell cycle, proliferation and differentiation of cancer cells were regulated by MYC. Abrogating MYC oncogenic function was one of targeted therapies for cancer. SE-lncRNAs transcritped from MYC SE locations and interacted with promoter of MYC to increase its expression in CRC. Therefore, SE-lncRNAs played a crucial role in the aggressive behavior of CRC cancer cells and could be a novel target.

### Stomach adenocarcinoma (STAD)

By analyzing H3K27ac ChIP-seq datasets from 11 STAD tissue and two cell lines and utilization of six algorithms (ImmuncellAI, CIBERSORT, EPIC, quantiSeq, TIMER, and xCELL), researchers identified the significantly dysregulated SE-associated lncRNAs that were strongly correlated with immune cell infiltration [31–33]. The expression of SE-lncRNA TM4SF1-AS1 was found negatively correlated with the proportion of CD8^+^ T cells in STAD. TM4SF1-AS1 suppressed T cell-mediated immune killing function and predicted immune response to anti-PD1 therapy [34]. SE-lncRNAs were involved not only in the proliferation and migration of cancer cells, but also in immune microenvironment.

### Breast cancer (BRCA)

To explore differentially expressed SE-lncRNAs which can identify mechanisms for ductal carcinoma in situ (DCIS) to invasive ductal carcinoma (IDC) progression, Ropri et al. integrally analyzed the enhancer loci with global expression of SE-lncRNAs in the MCF10A progression series. They screened out SE-lncRNAs RP11-379F4.4 and RP11-465B22.8 as potential markers through regulation of the expression of their neighboring genes (RARRES1 and miR-200b, respectively). Comparison analysis of acquired/lost super-enhancer regions classified in 47 ER positive patients, 10 triple negative breast cancer (TNBC) patients, and 11 TNBC cell lines revealed critically acquired pathways including STAT signaling and NF-kB signaling [35].

By global analysis of TCGA data, RNA-seq and ChIP-seq data, SE-lncRNA DSCAM-AS1 was identified regulated by transcription factor FOXA1, and DSCAM-AS1 was highly expressed and associated with poor prognosis in BRCA. FOXA1 promoted the transcription of DSCAM-AS1 through its two SEs location. Meanwhile, DSCAM-AS1 interacted with YBX1 to promote the expression of FOXA1 and ER, forming a positive feedback loop [36].

Interestingly, based on analysis of H3K27ac enrichment in hormone-deprived MCF-7 cells, Miano et al. defined a set of SEs occupied by apoERα, including one mapped in proximity of the DSCAM-AS1 lncRNA gene. They validated the enrichment of apoERα, p300, GATA3, FoxM1 and CTCF at both DSCAM-AS1 TSS (Transcription Start Site) and at its associated SE by ChIP-qPCR. Furthermore, by analyzing MCF-7 ChIA-PET data and 3 C assays, they confirmed long range chromatin interaction between the SE and the DSCAM-AS1 TSS [37].

By analyzing the FOXA1 ChIP-seq data and RNA-seq data in tamoxifen sensitive MCF7 and tamoxifen resistant MCF7/TamR cells, Zhang et al. identified 1003 super enhancer associated protein coding genes and five SE-lncRNAs (ATP1A1 − AS1, CASC11, CASC15, KCTD21 − AS1, LINC00885) related with tamoxifen resistance. However, only ATP1A1 − AS1 indicated high survival probability, while other three SE-lncRNAs (CASC15, KCTD21 − AS1, LINC00885) showed no significant prognosis value. Moreover, ATP1A1 − AS1 was low expression in MCF7/TamR cells and may play a crucial role in tamoxifen resistance [38].

BRCA was the most frequently diagnosed cancer in women. There were many valuable prognostic biomarkers in BRCA, such as PR, ER and Her2. Whether SE-lncRNAs could be regarded as promising therapeutic target of BRCA remained to be proved.

### Prostate cancer

Androgen receptor (AR)-negative castration-resistant prostate cancer (CRPC) was highly aggressive and resistant to most of the current therapies. Bromodomain and extra terminal domain (BET) protein BRD4 bound to SEs that promoted high expression of oncogenes in many cancers. For example, BRD4 bound to SEs locus of LncRNA-MANCR, whose expression was markedly decreased by BRD4 inhibitor. SE-lncRNA MANCR knockdown led to suppressed migration and invasion of AR-negative CRPC PC3 cells [39].

Based on H3K27ac ChIP-seq in enzalutamide (Enz)-resistant CRPC cells, Wen et al. identified a group of SEs (size > 50 kb) that were abnormally activated in Enz-resistant CRPC cells and associated with enhanced transcription of a subset of oncogenes like CHPT1. Increased CHPT1 conferred CRPC resistance to Enz. The CHPT1 SE activity and CHPT1 gene expression was regulated by a SE-lncRNA (CHPT1-eRNA) which transcribed at CHPT1 enhancer and interacted with BRD4 [40].

In addition to regulation of the proliferation and immunity homeostasis, SE-lncRNAs played a crucial role in therapy resistance of cancer cells. Further studies are needed to prove SE-lncRNAs’ targetable value in therapy resistance.

### Bladder cancer (BLCA)

Based on analysis of SE-lncRNA microarray between bladder cancer cells and normal bladder epithelial cells, Wang et al. identified LINC00162 as the SE-lncRNA with highest fold change value. In the range of 250KB upstream and downstream of LINC00162, PTTG1IP was bounded and pulled down by LINC00162 probes, indicating that PTTG1IP was the neighboring gene. With the RNA pull-down, protein purification and mass spectrometry analysis, the researcher found that THRAP3 interacted with LINC00162 and PTTG1IP. LINC00162 suppressed PTTG1IP expression through binding THRAP3 to promote progression of BLCA [41]. SE-lncRNAs, as a bridge, dragged transcription factors or enzymes to to target genes, forming a positive or negative feedback loop.

### Glioma

SE-lncRNA LIMD1-AS1 was expressed at a significantly higher level in glioma than in normal brain tissue. LIMD1-AS1 knockdown inhibited the proliferation, colony formation, migration, and invasion of glioma cell. Mechanically, SE inhibition significantly decreased the expression of LIMD1-AS1 by attenuating MED1 recruitment to the SE of LIMD1-AS1. Most importantly, LIMD1-AS1 could directly bind to HSPA5, leading to the activation of interferon signaling [42]. The signal pathway of “MED1/SE-lncRNA (LIMD1-AS1)/HSPA5” made a profound impact on glioma progression.

A novel SE-lncRNA, TMEM44-AS1, was aberrantly expressed in glioma tissues correlated with malignant progression. On the one hand, TMEM44-AS1 activated Myc and EGR1/IL-6 signaling by directly bound to the SerpinB3. On the other hand, Myc directly bounded to the promoter and SE of TMEM44-AS1 through MED1 and sequentially increased its transcription, thus forming a positive feedback loop [43]. In conclusion, TMEM44-AS1 and Myc formed an infinite amplifier in glioma.

By integrative analysis of RNA-seq data and ChIP-seq data of glioma patient-derived glioma stem cells (GSCs), Yang et al. screened out 6 SE-lncRNA, of which LINC00945 was further verified for the high expression of LINC00945 was specific in glioma. Overexpression of LINC00945 promoted proliferation, EMT, migration, and invasion of glioma cells. Mechanistically, BRD4 mediated epigenetic activation of LINC00945 [44]. The study constructed the prognostic SE-lncRNA signature and provided a potential therapeutic target for glioma.

### Nasopharyngeal carcinoma (NPC)

An integrative analysis based on whole-transcriptome sequencing and ChIP-seq pinpointed SE as a key mechanism underlying the vulnerability of NPC cells to inhibitor of SE treatment. DNA-binding motif analysis within the SE segments suggested that several transcription factors (including ETS2, MAFK, and TEAD1) may help establish and maintain SE activity across the genome. The study identified a novel SE-assocatied oncogenic transcripts LncRNA TP53TG1, which was highly and specifically expressed in NPC and functionally promoted NPC malignant phenotypes [45].

Tan et al. utilized ChIP-Seq to identify metastasis-specific SEs in NPC and found that the SE of LOC100506178 existed only in metastatic NPC cells and powerfully aggravated NPC metastasis. This metastatic SE transcribed into lncRNA LOC100506178. Furthermore, SE lncRNA-LOC100506178 promoted the transcription of MICAL2 by specifically interacting with hnRNPK, which in combination with the promoter region of MICAL2, subsequently enhancing EMT process and accelerating the invasion and metastasis of NPC cells. SE-LncRNA LOC100506178 could be a novel prognostic biomarker and therapeutic target in NPC patients [46].

Based on intersection analysis of 1785 differentially expressed lncRNAs from a microarray of NPC patients with metastatic lymph nodes and primary tumours and 442 SE-lncRNAs (https://rnajournal.cshlp.org/content/23/11/1729.full), Hu et al. identified two SE-lncRNAs (SUCLG2-AS1 and FAM225B). However, only SUCLG2-AS1 was significantly upregulated in 60 NPC tumor tissues at different stages. Moreover, SUCLG2-AS1 promoted NPC malignant phenotypes. Mechanistically, the m6A modification promoted RNA stability of SUCLG2-AS1. SUCLG2-AS1 improved the expression of SOX2 via long-range chromatin loop formation, which via mediating CTCF occupied the SE and promoter region of SOX2 [47].

In general, SE-lncRNAs transcription from or interaction with SE locations, could regulate the expression or function of key genes or signaling pathways in cancer cells. As a signal blocker, targeting SE-lncRNAs could be one of the most promising targets for NPC therapy.

### SE inhibitors and their implications in cancers

**Fig. 2:**
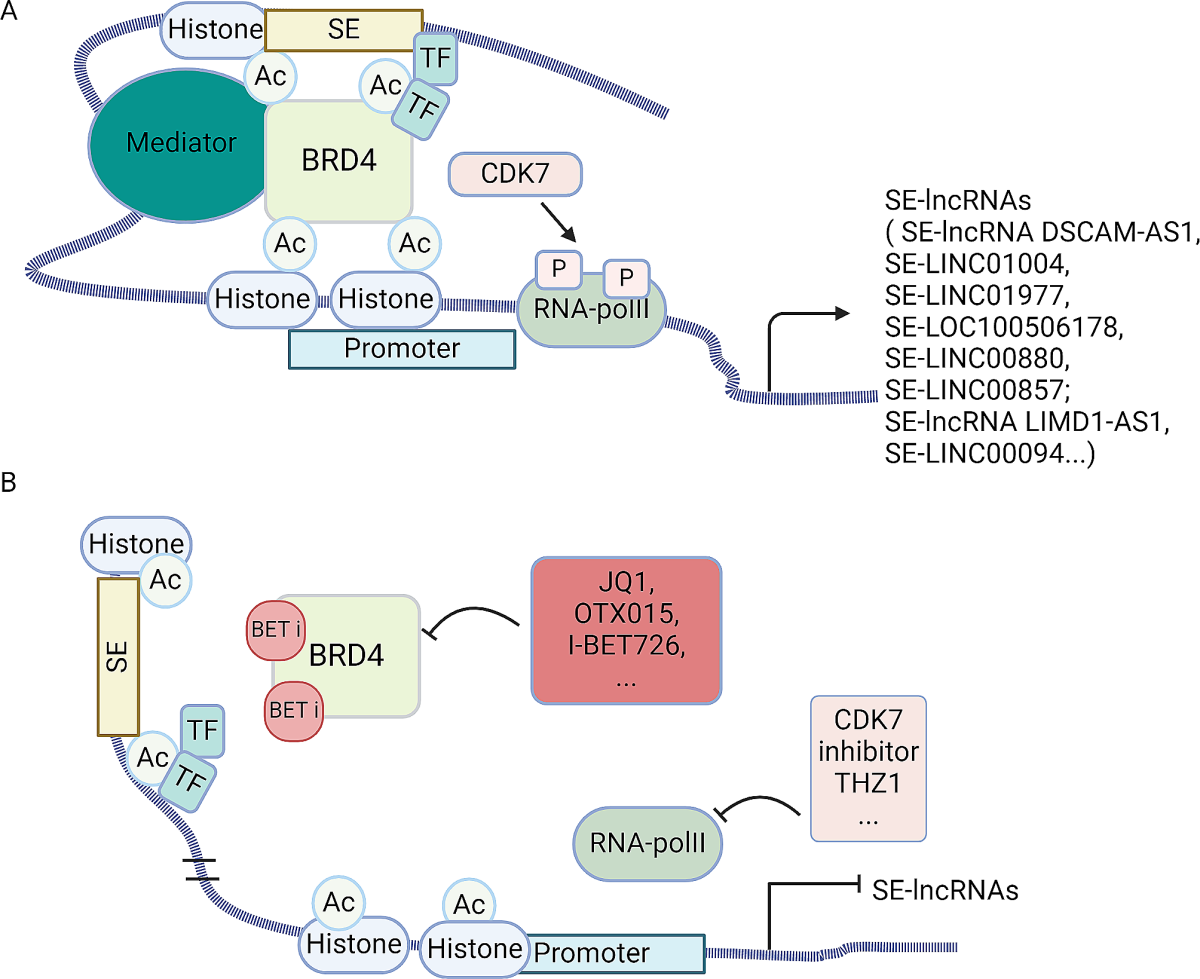
(**A**) Schematic representation of BRD4 and CDK7 acting on SE regions to promote the transcription of SE-lncRNAs. Super enhancers (SEs) consist of clusters of enhancers and harbor an unusually high density of transcription factors, mediator coactivators and epigenetic modifications. BRD4 recruits the mediator coactivators promoting the assembly of transcription factors, that forms a bridge between SE and Promoter, favoring and stabilizing the binding of RNA-PolII to promote the transcription of SE-lncRNAs. CDK7 dependent phosphorylation of RNA Pol II-carboxy terminal domain (CTD) on Ser-5 and Ser-7 facilitates transcription initiation. (**B**) The effect of inhibitors of BRD4 and CDK7 on oncogenes and SE-lnRNAs transcription. BET inhibitors compete with acetylated residues for releasing BRD4 from chromatin and disassembling the interaction between SE and Promoter, while CDK7 inhibitors reduce the phosphorylation of RNA Pol II, decreasing RNA-Pol II throughput and blocking transcription of SE-lncRNAs

Increasing studies showed that the repression of oncogenes via inhibiting SE complexes had become the most attractive target in cancer therapy [9, 48]. Several new drugs targeting SE complexes have been recently found to affect cellular transcription mechanisms, resulting in anti-tumor effects [49, 50]. Inhibitors of SE complexes, including BRD4 and CDKs, blocked transcription by inhibiting RNA polymerase II or affecting covalent histones modification (Fig. 2) [50–52]. We summarized the following SE complexes inhibitors in Table 2.

**Table 2:** the inhibitors of SEs

Inhibitor	Target	Cancer	Effects on tumor biology	Ref
JQ1	BRD4	PAAD, SCC, HCC, Lymphoma, OV, CRC, Gliomas, BRCA, LUAD, NPC	MicroRNAs, NOTCH signaling, NF-κB signaling, ALDH, KDM6A, MYC, FoxM1, MAPK signaling, SE-lncRNA DSCAM-AS1,LINC01004, LINC01977, LOC100506178	58–66, 27 17,25 36,46
AZD5153	BET	HCC, Leukemia	NAD + synthesis, MYC, MYB, b-catenin	67–68
OTX015 (MK-8628, Birabresib)	BET	Solid tumors, LUAD, Hematologic Malignancies,	MYCN, c-MYC, MAP3K8, SE-LINC00880	69–74 18
I-BET726	BRD4	Skin SCC, NB, Melanoma	c-Myc, Bcl-2, and cyclin D1,SphK1 Akt signaling, SE-LINC00857	75–76
PLX2853	BET	OV, Lymphomas	ARID1A, MYC	77–79
NHWD-870	BET	Solid tumor	CSF1, c-MYC	80
BMS986158	BET	PDAC, Rhabdomyosarcoma	Mitochondrial metabolism CRTFs	81–83
THZ1	CDK7	NPC, OSCC, Leukemia, SCC	PAK4, RUNX1, DNAJB1, SREBF2, YAP1, SE-lncRNA LIMD1-AS1, SE-LINC00094	91–95, 45, 23
THZ2	CDK7	Osteosarcoma TNBC	SE associated oncogenes “Achilles cluster”	96 97
SY-1365	CDK7	Leukemia, TNBC	MCL1	98
LDC4297	CDK7	PDAC	Transcription rates	89
BS-181	CDK7	Synovial sarcoma	3D spheroid formation and migration	99
ICEC0942	CDK7	BRCA	Synthetic lethality with tamoxifen	100
SY-5609	CDK7	Solid tumor	ClinicalTrials: NCT04247126	101
LY3405105	CDK7	Solid tumor	Stop at phase I trial	102
AZD4573	CDK9	Lymphoma	MYC, Mcl-1, JunB, PIM3	103
Alvocidib	CDK9	Leukemia/ Lymphoma	IRF4	105

BRD4 belongs to the BET family, which share a C-terminal extra terminal motif and two N -terminal tandem bromodomains [53]. BRD4 can interact with hyperacetylated histone regions on chromosome and accumulate on SE elements to promote gene transcription [54]. BET inhibitors compete with acetylated residues for releasing BRD4 from chromatin and disassembling the interaction between SE and promoter, reducing RNA-Pol II throughput and blocking transcription of key oncogene [55, 56].

MicroRNAs play a crucial role in cancer development [57]. SEs mark multiple miRNAs associated with cancer hallmarks [58, 59]. JQ1 inhibited SE-directed co-transcriptional pri-miRNA processing [58]. JQ1 could influence vital signal pathways in caner progression, such as inhibiting NOTCH signaling and NF-κB signaling, etc [27, 60]. . . In addition, JQ1 suppressed aldehyde dehydrogenase (ALDH) activity by abrogating BRD4-mediated ALDH1A1 expression through a SE element and its associated enhancer RNA [61]. KDM6A mutant pancreatic adenocarcinoma (PAAD) was sensitive to JQ1 [62]. Treatment with JQ1 in primary effusion lymphoma cell lines suppressed the expression of MYC and resulted in a genome-wide perturbation of MYC-dependent genes [63]. JQ1 repressed FoxM1 transcriptional program and induced pan-subtype cell-cycle arrest in ovarian cancer (OV) [64]. Combination of JQ1 with vemurafenib, a specific mutant BRAF inhibitor, suppressed cell growth by cell cycle arrest and induced apoptosis in the BRAFV600E-mutant CRC cells. Mechanistically, JQ1 repressed the vemurafenib-induced feedback activation of MAPK signaling pathway [65]. Likewise, due to reversal of inadvertent activation of detrimental SE programs in comparison with monotherapy, combinatorial treatment of CBP inhibitor ICG-001 with JQ1 was highly efficient in H3K27M-mutant diffuse intrinsic pontine gliomas (DIPG) [66]. In addition, JQ1 inhibited the expression of SE-lncRNA DSCAM-AS1 in BRCA, suppressing in vivo growth of xenograft tumors [36]. JQ1 also decreased SE-LINC01004 transcription and inhibited the proliferation and metastasis of HCC [25]. SE-LINC01977 was reduced with JQ1 in LUAD [17]. SE-LOC100506178 was inhibited by JQ1 treatment, leading to arrested invasion and metastasis of NPC cells [46].

AZD5153 is a bivalent BET bromodomain inhibitor [56, 67]. AZD5153 treatment upregulated NAMPT, whose product was the rate-limiting enzyme for NAD + synthesis [67]. AZD5153 acted synergistically with FK866, a potent NAMPT inhibitor, harnessing HCC cell proliferation and clonogenic survival. In response to AZD5153 treatment, NOTCH1-independent human T-cell leukemia exhibited a dose-dependent decrease in oncogenic drivers putatively under the control of super-enhancers such as MYC, MYB, and b-catenin [68].

OTX015 (known as MK-8628 and Birabresib) specifically binds to BRD2, BRD3, and BRD4, preventing BET proteins from binding to the chromatin, thus inhibiting gene transcription [69]. To date, OTX015 is under Phase 1 clinical trials in selected participants with advanced solid tumors and hematologic malignancies (ClinicalTrials.gov Identifier: NCT02698176, NCT02259114 and NCT02698189) [70, 71]. OTX015 specifically disrupted BRD4 binding and repressed transcription of MYCN, c-myc target genes and MAP3K8 expression [72–74]. In addition, OTX015 inhibited the expression of SE-lncRNA LINC00880 in LUAD [18].

I-BET726 is a novel BRD4 inhibitor. I-BET726 inhibited skin SCC cell growth in vitro and in vivo. In mechanism, I-BET726 not only downregulated BRD4-regulated proteins (c-Myc, Bcl-2, and cyclin D1), but also suppressed the activity of sphingosine kinase 1 (SphK1) and Akt signaling [75]. Targeting Myc with I-BET726 and JQ1 led to cell cycle arrest and induced cell immunogenicity of neuroblastoma (NB) and melanoma [76]. I-BET726 treatment decreased expression of SE-lncRNA LINC00857 and resulted in the deregulation of glutamine metabolism [30].

PLX2853 is an orally active, small-molecule inhibitor of BET bromodomain-mediated interactions that exhibits low nanomolar potency in blocking all four BET family members [77]. PLX2853 was effective in reversing platinum resistance in OV and generated synthetic lethality with ARID1A loss [78]. PLX2853 exhibited the enhanced responses of aggressive MYC-driven lymphomas [79]. Combination treatment of PLX2853 with trametinib in patients with uveal (eye) melanoma was under phase I/II clinical trial (ClinicalTrials.gov Identifier: NCT05677373).

NHWD-870, a BET inhibitor, could downregulate c-MYC and directly inhibite tumor cell proliferation. By inhibiting the tumoral expression and secretion of macrophage colony-stimulating factor CSF1, NHWD-870 blocked the proliferation of tumor associated macrophages (TAMs) [80].

BET inhibitor BMS-986,158 [81] disrupted mitochondrial function of cancer cells, leading to aberrant mitochondrial metabolism and stress via dysfunctional cellular respiration, proton leakage, and ATP production in pancreatic ductal adenocarcinoma (PDAC) [82]. In rhabdomyosarcoma, BMS-986,158 led to a decline in global transcription level and selective downregulation of core regulatory transcription factors (CRTFs) [83].

In addition, other studies have indicated that cyclin-dependent kinase 7 (CDK7) inhibitors have become one of the most powerful candidates to target oncogenic SEs [84]. CDK7, along with cyclin H and MAT1, forms the CDK-activating complex (CAK), which dirves progression via cell cycle and transcription initiation [85, 86]. Preclinical studies have shown that CDK7 inhibitors execute anti-cancer function partly depending on the repression of transcription, particularly transcription of super-enhancer-associated genes in cancer [87]. CDK7 inhibitors include THZ1, THZ2, SY-1365, LDC4297, BS-181, ICEC0942 (CT7001), SY-5609 and LY3405105, and some of them are now under Phase I/II clinical trials [84, 88–90]. As a covalent inhibitor of CDK7, THZ1 inhibited transcription by eliminating CDK7-dependent phosphorylation of RNA Pol II-carboxy terminal domain (CTD) on Ser-5 and Ser-7 [91, 92]. Recently, Nagaraja et al. reported that THZ1 treatment resulted in considerable disruption of global gene transcription in glioma cells, preferentially targeting SE-associated genes [93]. As mentioned above, oncogenic transcriptional amplification mediated by SE in NPC was vulnerable to THZ1 treatment [45]. THZ1 also was a highly potent anti-esophageal squamous cell carcinoma (OSCC) compound. Integrative analysis of both THZ1-sensitive and SE-associated transcripts identified several novel OSCC oncogenes, including PAK4, RUNX1, DNAJB1, SREBF2 and YAP1 [94]. In addition, THZ1 appeared to selectively target RUNX1 transcription via a ‘super- enhancer’ in T cell leukemia cells [95]. Furthermore, THZ1 reduced SE-lncRNA LIMD1-AS1 expression and inhibited the proliferation, colony formation, migration, and invasion of glioma [42]. Besides, the level of SE-LINC00094 also decreased with THZ1 treatment in SCC, leading to suppressed colony formation [23].

THZ2 was another CDK7 inhibitor [96]. Triple-negative breast cancer (TNBC)-specific genes was sensitive to THZ2 and frequently associated with SEs. Wang et al. concluded that CDK7 mediated transcriptional addiction to a vital cluster of genes of TNBC-specific genes, which was called ‘‘Achilles cluster’’. Therefore, THZ2 may be a promising therapy choice for TNBC [97]. Intriguingly, osteosarcoma SE-associated oncogenes were particularly vulnerable to THZ2 treatment [96].

SY-1365, a selective CDK7 inhibitor, was currently under clinical trials in patients with ovarian and breast cancer (NCT03134638). By decreasing MCL1 protein levels, SY-1365 inhibited the growth of different cancer types, including THP1 (Leukemia cell), HCC70 (BRCA cell) and RPE-hTERT (hTERT-immortalized cell lines) at nanomolar conceentartions in vitro. Moreover, by analyzing BCL2L1 in matched microarray datasets composed of 303 cell lines and RNA-seq datasets composed of 294 cell lines, the researchers found cancer cells with low BCL2L1 (BCL-XL) expression were more sensitive to SY-1365 [98].

LDC4297 limited the transcription rates and the phosphorylation of CAK in PDAC via non-covalently blocked ATP binding to CDK7 [89]. Similarly, via restriction of transcription activity, the CDK7 inhibitor BS-181 reduced 3D spheroid formation and migration of synovial sarcoma [99]. ICEC0942, a selective CDK7 inhibitor, was a prototype drug with potential utility that could be used as a single drug or in combination with tamoxifen for BRCA [100]. SY-5609, a highly potent inhibitor of CDK7 had already entered the clinic in 2020 (ClinicalTrials.gov Identifier: NCT04247126) [101]. LY3405105, a covalent inhibitor of CDK7, was under Phase I clinical trial evaluation. However, due to limited clinical activity, no further developments were conducted in advanced solid tumors [102].

CDK9 inhibitor also exerted antitumor activity by inhibiting RNA polymerase (Pol) II phosphorylation and suppressed SE-mediated, tumor-specific gene expression [103]. AZD4573, a highly selective and potent CDK9 inhibitor [104] which suppressed promoter activation and sustained reprograming of the SE landscape led to epigenetic remodeling. AZD4573 could downregulate multiple oncoproteins (MYC, Mcl-1, JunB, PIM3) and deregulate PI3K pathways in diffuse large B-cell lymphoma (DLBCL) [103]. Alvocidib was another CDK9 inhibitor that exerted antitumor activity by inhibiting IRF4 expression in adult T-cell leukemia/lymphoma via SE suppression [105].

## Conclusion and prospective

SE-lncRNAs play essential roles in tumorigenesis [95]. On the one hand, SE-lncRNAs can regulate key signaling pathways in tumor cells by influencing the transcription of oncogenes surrounding the certain chromatin locations [106]. On the other hand, SE-lncRNAs can recruit and transport necessary regulators (such as transcription factors and mediator complexes) to SEs, recombining chromatin organization by structural motifs and acting as spatial amplifiers for pivotal tissue-specific genes associated with SEs [107].

More and more technologies of high-throughput sequencing and next-generation sequencing (NGS) for exploring the SE-lncRNAs have emerged [108], including RNA-seq, ChIP-seq, DNase-seq, chromosome conformation capture (3 C) and SE-lncRNA microarrays [109]. Furthermore, the methods of FAIRE-seq, GRO-seq, ChIA-PET, ATAC-seq, STARR-seq and HiChIP were applied to identify the SE-lncRNAs [110]. In addtion, public databases like TRlnc (http://bio.licpathway.net/TRlnc) [111] and SELER (www.seler.cn) [15] were built for catching the crucial SE-lncRNAs in different cancers.

A growing number of inhibitors targeting SEs complexes have exhibited anti-cancer effects, of which some are already under clinical trials evaluation. Notebaly, the technology of CRISPR interference (CRISPRi), knocking out the SE region which transcribed carcinogenic SE-lncRNAs, can be regarded as a more accurate treatment [112]. In addition, wrapping SE-lncRNAs with nanomaterials may be a novel method for cancer therapy [113]. In summary, SE-lncRNAs is a promising hotpot in the research of carcinogenesis and progression. Targeting SEs would be a prospective therapy of cancer.

## Data Availability

No datasets were generated or analysed during the current study.
